# Phosphodiesterase-4 Inhibition Reduces Cutaneous Inflammation and IL-1β Expression in a Psoriasiform Mouse Model but Does Not Inhibit Inflammasome Activation

**DOI:** 10.3390/ijms222312878

**Published:** 2021-11-28

**Authors:** Barbara Meier-Schiesser, Mark Mellett, Marigdalia K. Ramirez-Fort, Julia-Tatjana Maul, Annika Klug, Nicola Winkelbeiner, Gabriele Fenini, Peter Schafer, Emmanuel Contassot, Lars E. French

**Affiliations:** 1 Department of Dermatology, University Hospital Zurich (USZ), Raemistrasse 100, 8091 Zurich, Switzerland; Mark.Mellett@usz.ch (M.M.); Julia-Tatjana-Maul@usz.ch (J.-T.M.); annika.klug@usz.ch (A.K.); nicola.winkelbeiner@usz.ch (N.W.); Gabriele.Fenini@usz.ch (G.F.); 2 BioFort, P.O. Box 1374, Guaynabo, PR 00970, USA; marigdalia@biofort.io; 3 Bristol Myers Squibb, 100 Nassau Park Blvd #300, Princeton, NJ 08540, USA; Peter.Schafer@bms.com; 4 Department of Biomedicine, Dermatology Department, Basel University Hospital, University of Basel, Hebelstrasse 20, 4031 Basel, Switzerland; emmanuel.contassot@unibas.ch; 5 Department of Dermatology, Ludwigs-Maximilians-University, Frauenlobstraße 9-11, 80337 Munich, Germany; Lars.French@med.uni-muenchen.de; 6 Dr. Philip Frost Department of Dermatology and Cutaneous Surgery, Miller School of Medicine, University of Miami, 1600 NW 10th Avenue, Miami, FL 33136, USA

**Keywords:** PDE4 inhibition, cytokines, inflammasome

## Abstract

Apremilast (Otezla^®^) is an oral small molecule phosphodiesterase 4 (PDE4) inhibitor approved for the treatment of psoriasis, psoriatic arthritis, and oral ulcers associated with Behçet’s disease. While PDE4 inhibition overall is mechanistically understood, the effect of apremilast on the innate immune response, particularly inflammasome activation, remains unknown. Here, we assessed the effect of apremilast in a psoriasis mouse model and primary human cells. Psoriatic lesion development in vivo was studied in K5.Stat3C transgenic mice treated with apremilast for 2 weeks, resulting in a moderate (2 mg/kg/day) to significant (6 mg/kg/day) resolution of inflamed plaques after 2-week treatment. Concomitantly, epidermal thickness dramatically decreased, the cutaneous immune cell infiltrate was reduced, and proinflammatory cytokines were significantly downregulated. Additionally, apremilast significantly inhibited lipopolysaccharide- or anti-CD3-induced expression of proinflammatory cytokines in peripheral mononuclear cells (PBMCs). Notably, inflammasome activation and secretion of IL-1β were not inhibited by apremilast in PBMCs and in human primary keratinocytes. Collectively, apremilast effectively alleviated the psoriatic phenotype of K5.Stat3 transgenic mice, further substantiating PDE4 inhibitor-efficiency in targeting key clinical, histopathological and inflammatory features of psoriasis. Despite lacking direct effect on inflammasome activation, reduced priming of inflammasome components upon apremilast treatment reflected the indirect benefit of PDE4 inhibition in reducing inflammation.

## 1. Introduction

Psoriasis is a common chronic inflammatory disease of the skin that is characterized by epidermal hyperplasia and hyperkeratosis. The most common form, psoriasis vulgaris, affects 2–3% of the world population, most frequently beginning in young adults and persisting throughout life [1]. Defective innate and adaptive immune responses are involved in the pathogenesis of psoriatic lesions [2]. A variety of therapeutic modalities are used in the context of intermittent or continuous management of psoriasis [3]. Standard treatment includes topical steroids, UV therapy [4], immunomodulators like methotrexate [5,6] and dimethylfumarate [7,8,9]. More recently, antibody-based targeted therapy directed against proinflammatory cytokines (e.g., TNFα, IL-17A and IL-23), has become part of the standard of care for patients with moderate to severe plaque psoriasis [10,11,12,13,14,15,16,17]. However, these biological therapies are not approved for patients with less severe psoriasis and have to be administered by regular subcutaneous or intravenous injection.

Apremilast (Otezla^®^) is a first-in-class oral small molecule phosphodiesterase 4 (PDE4) inhibitor approved for the treatment of psoriasis, psoriatic arthritis, and oral ulcers associated with Behçet’s Disease [18,19]. Cyclic nucleotide PDEs catalyze the hydrolysis of the phosphodiester bond in cyclic adenosine monophosphate (cAMP) or cyclic guanosine monophosphate (cGMP). Cyclic AMP is a key second messenger acting as an intrinsic modulator of inflammatory responses, exerting pleiotropic effects within a number of immune cells including T cells, dendritic cells and macrophages [20]. PDE4 is the predominant cAMP-selective PDE expressed in inflammatory cells [21]. Inhibition of PDE4 results in elevated intracellular cAMP levels, which, in turn, down-regulate cellular inflammatory responses by modulating the expression of inflammatory cytokines, such as TNFα, IL-23, IL-12 and IL-17A and by upregulating expression of the anti-inflammatory cytokine IL-10 [22,23].

In 2015, apremilast was approved by the European Medicines Agency for the treatment of moderate to severe plaque psoriasis. Apremilast clinical investigations took place in multiple Phase III and Phase IV clinical trials for the treatment of psoriasis, psoriatic arthritis and scalp psoriasis [24] as well as other chronic inflammatory diseases [25].

Interestingly, cAMP suppresses NLRP3 inflammasome activation in murine macrophages and was previously reported to bind to the nucleotide-binding domain of NLRP3, a common location of Cryopyrin-Associated Autoinflammatory Syndromes (CAPS)-associated mutations [26,27]. Prostaglandin E_2_ (PGE_2_) also inhibits NLRP3 inflammasome activation in human macrophages, an effect mediated by an increase in intracellular cAMP [28].

While the overall mechanism of action of PDE4 inhibition is understood, the effect of apremilast on innate immunity, notably inflammasome activation and regulation of IL-1β release, remains unknown. IL-1β is reported to play a role early in psoriasis disease development [29,30]. Therefore, to appraise the role of apremilast in vivo and on the innate immune system, we assessed the effect of apremilast in a transgenic mouse model of psoriasis and evaluated its effect on the expression of inflammatory cytokines, including IL-1β.

## 2. Results

To test the effect of apremilast in vivo, we used K5.Stat3C transgenic mice as a physiologically relevant psoriasiform disease model. These mice express constitutively active Stat3 (Stat3C) under the control of the keratin 5 (K5) promoter, ensuring expression in the basal layer of the epidermis. Due to this genetic modification, tape-stripping of mouse skin, the equivalent of the Koebner phenomenon in humans, results in micro- and macroscopic changes that are morphologically similar to psoriatic skin [31]. After tape-stripping, a total of 15 out of 20 (75%) mice developed psoriatic lesions that were suitable for further investigation.

**Figure 1:**
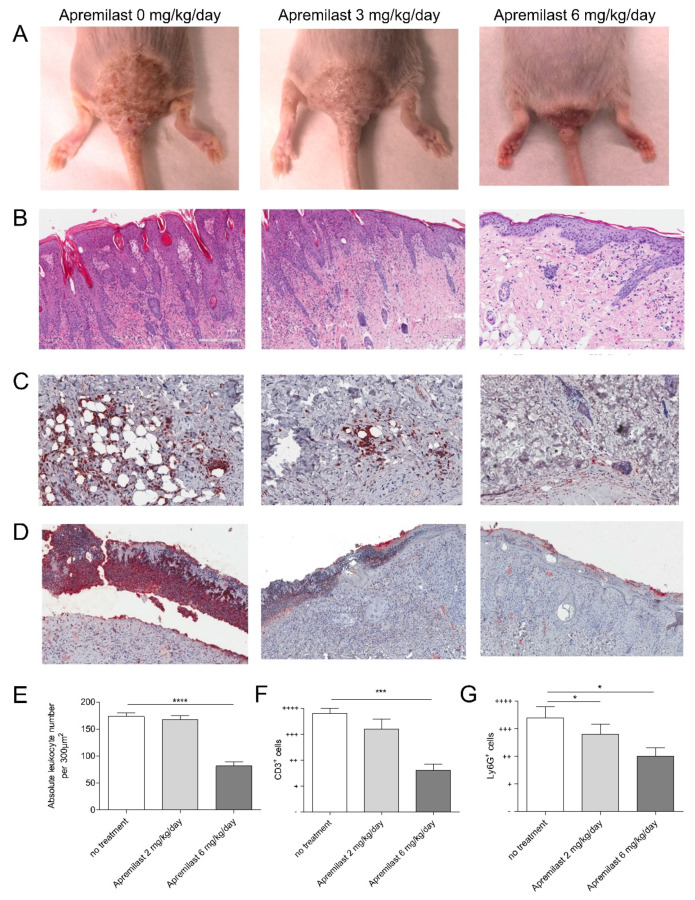
Heterozygous K5.Stat3C mice were tape-stripped for two weeks to induce psoriatic skin lesions. These mice were then either left untreated or treated with 2 mg/kg/day or 6 mg/kg/day of apremilast for two weeks. Macroscopic appearance (**A**) and histology (**B**) are shown. After Hematoxilin Eosin coloration of skin sections, total leukocytes were counted in 300 µm^2^ fields (**E**). Skin sections were then stained with an anti-mouse CD3 (**C**) and with an anti-Ly6G (**D**) antibody and semi-quantified (**F**,**G**). Expression levels were qualified as very strong (++++), strong (+++), moderate (++), weak (+) or absent (0). Shown are the mean +/− SEM of leukocyte cell counts and CD3+ and Ly6G+ expression levels in 5 mice/condition. ****: *p* < 0.0001, ***: *p* < 0.001, *: *p* < 0.05.

Five mice with psoriatic lesions were administered apremilast at 2 mg/kg/day, five mice received 6 mg/kg/day and five mice were left untreated as positive controls. Five mice without prior tape-stripping and consecutive development of psoriatic lesions served as negative control mice. Treatment with apremilast at 2 mg/kg/day and 6 mg/kg/day resulted in moderate and significant resolution of inflamed plaques after 2 weeks of treatment, respectively (Figure 1A). Histological examination of psoriatic lesions revealed dramatically decreased epidermal thickening and a reduced leukocytic dermal infiltrate in mice treated with the higher dose of apremilast, whereas a minimal effect was seen in the skin of mice treated with 2 mg/kg/day (Figure 1B,E). Immunohistochemical analysis revealed a reduced infiltration of CD3^+^ lymphocytes and Ly6G^+^ neutrophils upon treatment with apremilast at 2 mg/kg/day, which was more pronounced with 6 mg/kg/day (Figure 1C,D,F,G).

**Figure 2:**
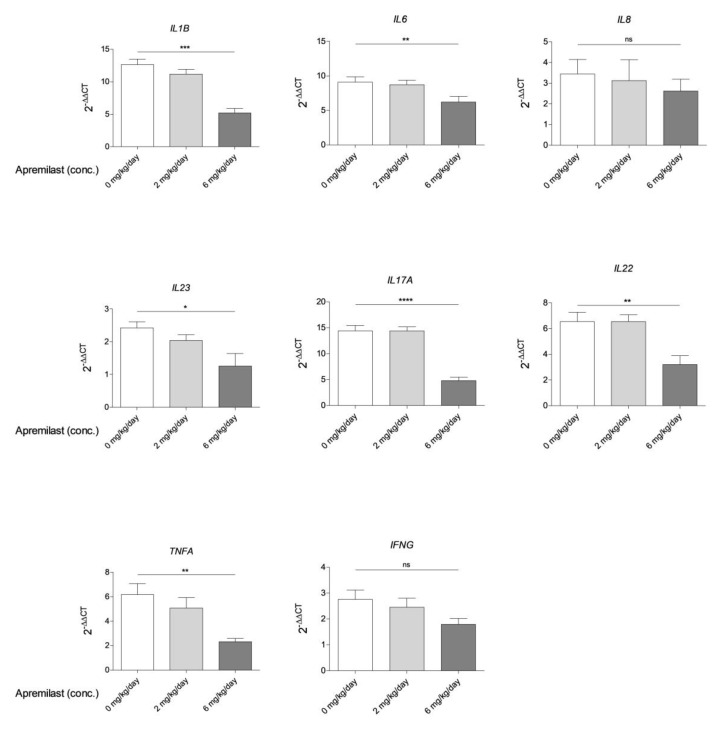
Quantitative PCR was performed on lesional skin samples of heterozygous K5.Stat3C mice either left untreated or treated with 2 mg/kg/day or 6 mg/kg/day apremilast for two weeks. qPCR was performed with primers for the indicated murine cytokines. Non-lesional skin from K5.Stat3C mice served as an internal control (2^−^^ᴧᴧCT^ = 1). Shown is the mean ± SEM of 5 mice/condition. Statistical analysis was performed using One-way Anova followed by Tukey’s posthoc test. ****: *p* < 0.0001, ***: *p* < 0.001, **: *p* < 0.01, *: *p* < 0.05, ns: *p* ≥ 0.05.

Real-time quantitative PCR (qPCR) analysis of lesional skin indicated that 6 mg/kg/day apremilast treatment led to a significant downregulation of *IL1B*, *IL6*, *IL23*, *IL17A*, *IL22*, and *TNFA* transcript levels (*n* = 5) when compared to untreated mice (Figure 2). *IL8* and *IFNG* were downregulated upon treatment, however, not significantly. No significant downregulation of the above cytokines could be detected in mice treated with 2 mg/kg/day of the drug.

**Figure 3:**
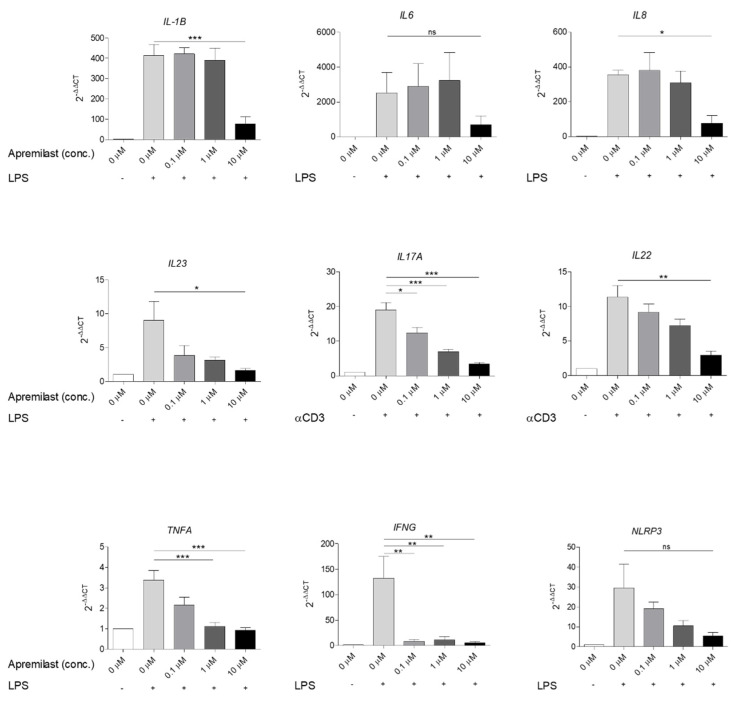
Human PBMCs were treated with 0.1 µM, 1 µM or 10 µM apremilast for one hour followed by upLPS (100 ng/mL) or anti-CD3 stimulation (50 μg/mL). After overnight incubation at 37 °C, cells were harvested for RNA isolation. The mRNA expression levels were detected using qPCR with primers for *IL1B, IL6, IL8, IL23, IL17A, IL22, TNFA* and *IFNG.* Shown is the mean ± SEM of 4 healthy individuals. Statistical analysis was performed using One-way Anova followed by Tukey’s posthoc test. ***: *p* < 0.001, **: *p* < 0.01, *: *p* < 0.05, ns: *p* ≥ 0.05, +: with, -: without.

To assess the effect of apremilast on the response of human immune cells, we stimulated peripheral mononuclear cells (PBMCs) with 100 ng/mL ultra-pure lipopolysaccharide (upLPS) or anti-CD3 overnight to induce the expression of pro-inflammatory cytokines including *IL1B*, *IL6*, and chemokine *IL8*, as well as *IL23*, *IL17A*, *IL22*, *TNFA* and *IFNG.* In PBMCs, apremilast at a concentration of 10 µM significantly inhibited the expression of *IL1B*, *IL8*, *IL23*, *IL17A*, *IL22*, *TNFA* and *IFNG* after stimulation with upLPS (*IL1B*, *IL8*, *IL23*, *TNFA* and *IFNG*) or anti-CD3 (*IL17A*, *IL22*). *IL6* and *NLRP3* showed a clear trend of decreased expression, however, this was not statistically significant. Moreover, *IL17A*, *TNFA* and *IFNG* expression was also significantly decreased at concentrations of 0.1 µM and 1 µM apremilast. The other cytokines showed only a trend towards a decrease in expression at apremilast concentrations of 0.1 µM and 1 µM (Figure 3).

**Figure 4:**
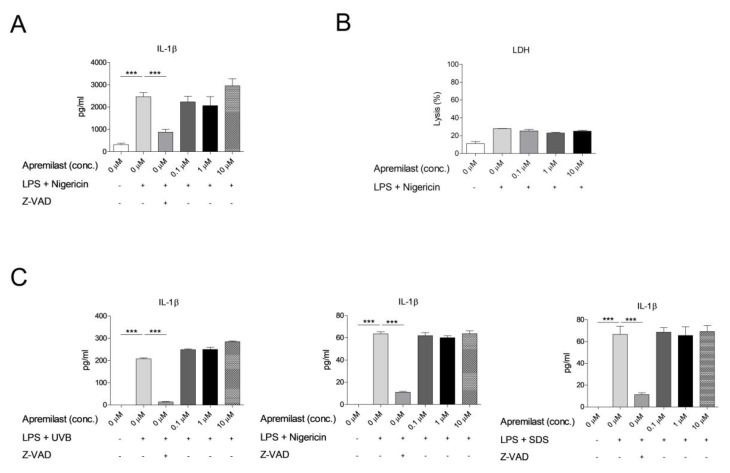
(**A**) Human PBMCs were stimulated with upLPS overnight, followed by treatment with Z-VAD, or 0.1 µM, 1 µM or 10 µM apremilast for one hour before nigericin was added. (**B**) Cell death was measured using a lactate dehydrogenase (LDH) assay after stimulation of PBMCs with upLPS + Nigericin and apremilast at different doses. (**C**) Human primary keratinocytes were cultured for 24 h before treatment with upLPS overnight, followed by treatment with Z-VAD, or 0.1 µM, 1 µM or 10 µM apremilast two hrs before nigericin or SDS treatment or UVB-irradiation (0.5 J/cm^2^). After five hrs incubation at 37 °C, the supernatant was collected and IL-1β ELISA was performed. Mean ± SEM of four different healthy individuals is shown.***: *p* < 0.001, +: with, -: without.

As *IL1B* expression could be blocked with apremilast at a concentration of 10 µM, we aimed to investigate the ability of apremilast to suppress the secretion of active IL-1β. Indeed, IL-1β is first produced in an inactive form upon NF-κB pathway activation (signal 1) and needs to be cleaved to become biologically active (signal 2). This cleavage is performed by cytosolic multimeric immune complexes, called inflammasomes, which recruit the adaptor molecule ASC and inflammatory caspase, Caspase-1. Recruitment of Caspase-1 facilitates its cleavage and active Caspase-1 can cleave pro-IL-1β and IL-18 into their mature bio-active forms (reviewed in [32]). Inflammasomes are activated upon cellular infection or stress and lead to the maturation and release of IL-1β which in turn engages and amplifies innate immune defenses [33]. Due to the potent physiological effects of IL-1β, inflammasome activation is tightly regulated and “priming” or transcription of inflammasome components, such as NLRP3 and pro-IL-1β itself, is required before inflammasome activation. Therefore, to induce the secretion of active IL-1β, human PBMCs were stimulated with the inflammasome activator nigericin (signal 2) after overnight exposure to upLPS (signal 1). To assess the ability of apremilast to inhibit inflammasome activation, upLPS-stimulated cells were treated with either 0.1 µM, 1 µM, or 10 µM apremilast or the pan-caspase inhibitor Z-VAD two hours prior to stimulation with nigericin (Figure 4A). Likewise, human primary keratinocytes (KCs) were stimulated with the NLRP1 inflammasome activators UVB, and the NLRP3 activators sodium dodecyl sulphate (SDS) and nigericin one hour after treatment with either apremilast or Z-VAD (Figure 4B). Secreted IL-1β was measured in the supernatant of PBMCs and KCs, respectively. In this experimental setup, the secretion of IL-1β was not inhibited by apremilast at concentrations of 0.1 µM, 1 µM and 10 µM both in PBMCs and in KCs under different conditions as revealed by ELISA on culture supernatant (Figure 4A,B). Similarly, apremilast did not inhibit upLPS and nigericin-induced pyroptosis in PBMCs, as indicated by lactate dehydrogenase (LDH) release (Figure 4B). Apremilast also did not induce an increase in cell death, even at higher concentrations (Figure 4B). These results suggest that apremilast inhibits the production of the pro-form of IL-1β but is unable to inhibit inflammasome activation as evidenced by the unaffected release of mature IL-1β.

## 3. Discussion

Apremilast is a small molecule inhibitor of PDE4, which deactivates cAMP, an intrinsic modulator of inflammatory responses. The drug is a registered orally-administered systemic therapy for psoriasis and psoriatic arthritis [24,34,35], and a newly registered systemic treatment of oral ulcers in Behçet’s disease [25].

Here, we demonstrate the efficacy of PDE4 inhibition on the reduction of the psoriatic phenotype of K5.Stat3 transgenic mice. Apremilast administered at a dose of 6 mg/kg per day reduced psoriatic plaques macroscopically and histologically. This effect was dose-dependent as mice treated with 2 mg/kg per day showed only a moderate improvement. Moreover, the gene expression of the proinflammatory cytokines *IL1B*, *IL6*, *IL23*, *IL17A*, *IL22*, *TNFA* and *IFNG* in skin lesions was significantly reduced after 2 weeks of apremilast treatment at 6 mg/kg/day. Although not significant, *IL8* and *IFNG* expression were also decreased in these conditions. Similar observations were reported in the beige-SCID mouse human skin/psoriatic NK cell xenograft model where 5 mg/kg per day led to significantly lower epidermal thickening, decreased keratinocyte proliferation and reduction of *TNFA* expression [19]. Furthermore, in a murine model of colitis, a significantly lower level of IL-18 was found after apremilast treatment, which is consistent with our data [36]. Nevertheless, direct translation from animal models into human disease always has its limitations [37]. Models like the K5.Stat3C transgenic mice recapitulate many of the key features of psoriasis [31], however, no mouse model completely mirrors the systemic effects of human psoriasis and its comorbidities, thus observations are limited to a “snapshot” of the human condition. The key cytokines in human disease are also significantly highly expressed in the skin of this model, including *TNFA*, *IFNG*, *IL17A*, *IL23*, *IL22* and *IL1B*. Apremilast reduces the expression of *TNFA*, *IL-23*, *IL12* and *IL17A* in human disease and since this is the case in this model, it would suggest that it is a good model for assessing apremilast effects in vivo. Another interesting aspect of the K5.Stat3C transgenic mice is their reactivity to trauma to the skin, which mirrors the human Koebner reaction.

Interestingly, reduced expression of vitamin D receptors (VDR) has been associated with psoriasis [38,39,40] and a lack of VDR has been shown to activate STAT3 signaling in retinal ganglion cell damage. It is interesting to speculate that diminished VDR might also activate excessive STAT3 in keratinocytes. VDR appears to be associated with the expression of critical tight-junction proteins and reduced integrity of tight junction complex in psoriatic skin might be due to a lack of VDR expression. Significantly, topical calcipotriol inhibits the IL-23/IL-17 axis and IL-36 inflammatory loop in the imiquimod psoriasiform mouse model via VDR signaling on keratinocytes [41].

In human PBMCs stimulated with the TLR4 agonist upLPS or with the T cell stimulus anti-CD3, apremilast treatment led to a reduction of *IL1B*, *IL6*, *IL8*, *IL23*, *IL17A*, *IL22*, *TNFA* and *IFNG* expression confirming previous reports [42,43]. Notably, apremilast was able to block the expression of innate cytokines, which is indicative of an inhibition of NF-κB transcriptional activity and is consistent with previous reports [42]. As the transcription of *IL1B* was decreased by apremilast treatment both in the mouse model and in human PBMCs, we aimed to determine whether apremilast could also inhibit the secretion of active IL-1β through inhibition of the inflammasome. In contrast to the transcriptional data, apremilast failed to prevent the secretion of IL-1β in response to inflammasome activators in both human PBMCs and keratinocytes. Interestingly, apremilast reduced expression levels of *NLRP3* in PBMCs in a dose-dependent manner. Moreover, the reduction of LPS-induced cytokine expression suggests that apremilast has an inhibitory effect on the NF-κB pathway, involved in LPS-TLR4 signaling. Due to the regulatory effect on NF-κB, the expression of inflammasome components, such as NLRP3 was reduced by apremilast treatment. Given the long-term development of skin lesions and the apremilast-induced inhibition of other inflammatory cytokines, IL-1β reduction in treated mice could also be an indirect effect. It is also plausible that reduction of TNFα and IL-17A expression could have a dramatic impact on inflammasome activation and IL-1β levels in vivo.

To our knowledge, the effects of PDE4 inhibition by apremilast in the context of inflammasome activation in human cells have not been reported. Previously, the expression levels of mRNA encoding inflammasome components were analyzed in a murine model of colitis and significantly lower levels of inflammasome-encoding gene expression including *IL18* and *NLRP3* were found in response to PDE4 inhibition [36]. This corroborates our findings that apremilast exerts an inhibitory effect on NF-κB signaling, therefore, subsequently impacting inflammasome expression and priming rather than its activation.

Collectively, we report the efficacy of apremilast in the reduction of epidermal thickening, leukocyte infiltration and expression of key proinflammatory cytokines in the K5.Stat3 mouse model of psoriasis, further supporting the ability of this PDE4 inhibitor to efficiently target key clinical, histopathological and inflammatory features of psoriasis. While no direct effect of apremilast on inflammasome activation was observed, priming of inflammasome components was reduced, highlighting an additional, albeit indirect, benefit of PDE4 inhibition in reducing inflammation.

## 4. Materials and Methods

### 4.1. Mice

K5.Stat3C transgenic mice were chosen as a physiologically relevant model of psoriasis because they express a constitutively active Stat3 (Stat3C) under the control of the keratin 5 (K5) promoter, which results in a micro- and macroscopic psoriasis phenotype [31]. The Koebner phenomenon is effectively reproduced by tape-stripping K5.Stat3C transgenic mice. All the experiments were approved by the local Animal Protection Authorities.

### 4.2. Human Blood Sampling

All human samples (healthy individuals) were collected with informed written consent upon approval of Local Ethical Committees and were conducted according to the Declaration of Helsinki Principals.

### 4.3. Apremilast Treatment of Mice

Twenty heterozygous K5.Stat3 mice were tape-stripped for three consecutive days to obtain psoriatic skin lesions in 15 mice. A latency period of two weeks was permitted before mice were randomized into untreated, 2 mg/kg apremilast or 6 mg/kg apremilast per day groups (five mice per group). These doses are approximately 25% and 75% of the maximum recommended human therapeutic dose [37]. Apremilast was administered in the drinking water. Dose calculations were made based on the average daily water intake of each mouse.

### 4.4. RNA Isolation, cDNA Synthesis and Real-Time qPCR

RNA was isolated from mouse skin and human PBMCs, respectively, using the RNeasy Fibrous Kit (Qiagen, Hilden, Germany) according to manufacturer’s instructions, followed by cDNA synthesis by reverse transcription using the Revert Aid First Strand cDNA Kit (Thermo Scientific, Waltham, MA, USA). Samples were incubated for 5 min at 25 °C, 60 min at 42 °C and 5 min at 70 °C. The real-time qPCR was performed with a cDNA primer pair, designed to a fragment of ~150 bp in length, flanking the intron-exon border of the gene of interest. RPL27 served as an internal reference gene. Quantitative PCR was performed using primers for cytokine-encoding genes including *IL1B*, *IL6*, *IL8*, *IL23*, *IL17A*, *IL22*, *TNFA* and *IFNG* (mouse and human, respectively).

### 4.5. Immunohistochemistry

Immunohistochemical stainings were performed as previously described [31] using an anti-CD3 (Abcam, Cambridge, UK) and an anti-Ly6G (Biolegend, San Diego, CA, USA) antibody.

### 4.6. Culture of PBMCs

Human blood from healthy controls was obtained from the Dermatology department, University Hospital Zurich, Switzerland. PBMCs were isolated using the Ficoll-Paque density gradient technique (Ficoll-Paque, Pharmacia, Glattbrugg, Switzerland), as previously described [38]. Cells were counted and plated in 96-well plates in complete RPMI 1640 medium at 1 × 106 cells/mL (100 µL per well). The cells were maintained in culture for 2 h before investigational use. PBMCs were stimulated with 100 ng/mL ultrapure lipopolysaccharide (upLPS, *E. coli* 0111:B4; InvivoGen, San Diego, CA, USA) or 50 μg/mL anti-CD3 (Biolegend, San Diego, CA, USA) to induce expression of pro-inflammatory cytokines.

### 4.7. Culture of Human Primary Keratinocytes

Human primary foreskin keratinocytes (KC) were isolated as previously described [20] and passaged in human keratinocyte serum-free medium supplemented with EGF and BPE (Gibco BRL; Paisley, Scotland). Cells were plated after 3 passages, into 6-well plates (2.5 mL × 0.150 mio/mL).

### 4.8. Inflammasome Activation

PBMCs and keratinocytes were incubated at 37 °C in a humidified atmosphere of 5% CO_2_ for 2 and 24 h, respectively. PBMCs were then primed with upLPS (100 ng/mL) for 16 h. Apremilast or 1 µM Z-VAD (Selleck Chemicals, Houston, TX, USA) treatment was applied for 1 h before stimulation with 1 µM nigericin (Selleck Chemicals, Houston, TX, USA) for PBMCs or with 86.4 mJ/cm^2^ UVB (UV802L; Waldmann, Villingen-Schwenningen, Germany), nigericin [39] or SDS (15 µg/mL) for keratinocytes. After 6 h of incubation at 37 °C, the supernatant was collected.

### 4.9. Analysis of Supernatant

Cell culture supernatants were collected at the indicated time points and analyzed for the presence of IL-1β by enzyme-linked immunosorbent assay (ELISA). IL-1β ELISA (R&D, Minneapolis, MN, USA) was performed according to the manufacturer’s instructions. Cell death was measured by LDH release.

## Data Availability

Not applicable.
